# Efficacy of repetitive transcranial magnetic stimulation (rTMS) for reducing consumption in patients with alcohol use disorders (ALCOSTIM): study protocol for a randomized controlled trial

**DOI:** 10.1186/s13063-021-05940-z

**Published:** 2022-01-12

**Authors:** Benjamin Petit, Agnès Soudry-Faure, Ludovic Jeanjean, Jack Foucher, Laurence Lalanne, Maud Carpentier, Lysiane Jonval, Coralie Allard, Mathilde Ravier, Amine Ben Mohamed, Vincent Meille, Benoit Trojak

**Affiliations:** 1 Department of Addictology, University Hospital of Dijon, 14 rue Paul Gaffarel, B.P. 77908, 21079 Dijon Cedex, France; 2 UFR des Sciences de Santé, Université de Bourgogne, Dijon, France; 3 Unité de Soutien Méthodologique à la Recherche, Délégation à la Recherche et à l’Innovation (DRCI), University Hospital of Dijon, 1 boulevard Jeanne d’Arc BP 77 908, 21 079 Dijon Cedex, France; 4 UMR CNRS 7357 iCube, FMTS (Fédération de Médecine Translationnelle de Strasbourg), Université de Strasbourg, Strasbourg, France; 5 Centre de neuroModulation Non-Invasive de Strasbourg – CEMNIS, Hôpitaux Universitaires de Strasbourg, Hôpital Civil, 1 place de l’Hopital, BP426, 67091 Strasbourg Cedex, France; 6 INSERM 1114, Department of Psychiatry and Addictology, University Hospital of Strasbourg, Fédération de Médecine Translationnelle de Strasbourg (FMTS), 67000 Strasbourg, France; 7 Department of Psychiatry and Addictology, University Hospital of Strasbourg, Fédération de Médecine Translationnelle de Strasbourg (FMTS), 67000 Strasbourg, France; 8 Pôle Psychiatrie, Santé Mentale et Addictologie, Clinique de psychiatrie, Hôpitaux Universitaires de Strasbourg, 1 place de l’hôpital, BP 426, F-67091 Strasbourg Cedex, France; 9 Délégation à la Recherche et à l’Innovation (DRCI), University Hospital of Dijon, 1 boulevard Jeanne d’Arc – BP 77 908, 21079 Dijon Cedex, France; 10 INSERM UMR1093-CAPS, Université Bourgogne Franche-Comté, UFR des Sciences du Sport, Dijon, France

**Keywords:** Addiction, Alcohol use disorder, Reduction, Repetitive magnetic transcranial stimulation, Non-invasive brain stimulation

## Abstract

**Background:**

The number of people with an alcohol use disorder (AUD) was recently estimated to be 63.5 million worldwide. The global burden of disease and injury attributable to alcohol is considerable: about 3 million deaths, namely one in 20, were caused by alcohol in 2015. At the same time, AUD remains seriously undertreated.

In this context, alternative or adjunctive therapies such as brain stimulation could play an important role. The early results of studies using repetitive transcranial magnetic stimulation (rTMS) suggest that stimulations delivered to the dorsolateral prefrontal cortex significantly reduce cravings and improve decision-making processes in various addictive disorders. We therefore hypothesize that rTMS could lead to a decrease in alcohol consumption in patients with AUD.

**Methods/design:**

We report the protocol of a randomized, double-blind, placebo-controlled, parallel-group trial to evaluate the efficacy of rTMS on alcohol reduction in individuals diagnosed with AUD. The study will be conducted in 2 centers in France. Altogether, 144 subjects older than 18 years and diagnosed with AUD will be randomized to receive 5 consecutive twice-daily sessions of either active or sham rTMS (10 Hz over the right DLPFC, 2000 pulses per day). The main outcomes of the study will be changes in alcohol consumption within the 4 weeks after the rTMS sessions. Secondary outcome measures will include changes in alcohol consumption within the 24 weeks, alcohol cravings, clinical and biological improvements, effects on mood and quality of life, and cognitive and safety assessments, and, for smokers, an assessment of the effects of rTMS on tobacco consumption.

**Discussion:**

Several studies have observed a beneficial effect of rTMS on substance use disorders by reducing craving, impulsivity, and risk-taking behavior and suggest that rTMS may be a promising treatment in addiction. However, to date, no studies have included sufficiently large samples and sufficient follow-up to confirm this hypothesis. The results from this large randomized controlled trial will give a better overview of the therapeutic potential of rTMS in AUD.

**Trial registration:**

ClinicalTrials.gov NCT04773691. Registered on 26 February 2021

https://clinicaltrials.gov/ct2/show/NCT04773691?term=trojak&draw=2&rank=5.

## Background

Alcohol use disorder (AUD) is considered a major public health problem in Western societies [1]. In 2015, the number of people suffering from an alcohol use disorder was estimated at 63.5 million, namely 843 per 100,000 people, which represents 8.6% of men and 1.7% of women [2, 3]. The global burden of disease and injury attributable to alcohol is considerable: in 2016, about 3 million deaths (approximately 1/20) worldwide were caused by alcohol [4]. In the EU in 2015, alcohol was responsible for 108 deaths per 100,000 people [2]. Because alcohol is implicated in more than 60 diseases (i.e., vascular, endocrine, and neurological diseases, cancer, etc.), and many non-fatal injuries early in life, the disability-adjusted life years (DALYs) are even higher: in 2016, 5.1% of all DALYs, i.e. 132.6 million DALYs, were caused by alcohol (men: 106.5 million; women: 26.1 million) [2, 4].

At the same time, alcohol use disorders remain seriously undertreated. There is a large treatment gap, given that only around 20% of people in Europe and the USA with a diagnosed AUD actually receive any treatment [5–8]. One of the main reasons is that these patients are not ready to stop drinking and thus are not attracted to the abstinence goals anticipated by the current psychosocial and pharmacological treatments [9]. New treatment strategies that aim to reduce alcohol consumption could make it much easier for patients to ask for help. Thus, new treatments supporting this strategy are required to improve the care of patients suffering from AUD.

In this context, alternative or adjunctive therapies such as repetitive transcranial magnetic stimulation (rTMS) and transcranial direct current stimulation (tDCS), two non-invasive brain stimulation techniques, may play a prominent role. They focally modulate the neuronal excitability of superficial brain regions, and even deeper structures thanks to brain connectivity [10]. Indeed, neuroimaging studies have identified changes in the prefrontal regions of patients diagnosed with addictive disorders, in particular in the dorsolateral prefrontal cortex (DLPFC) [11]. These brain changes were associated with cravings, manifested by an intense desire or urge to consume a drug, and with impaired inhibitory control [10, 12, 13]. Overall, the early results of studies using rTMS and tDCS applied to the DLPFC found a significantly reduced craving levels in various addictive disorders (tobacco, alcohol, marijuana, and methamphetamines) [14–18]. These results were consolidated by a meta-analysis that included 17 studies [11]. In this meta-analysis, random-effects analysis revealed a pooled standardized effect size (Hedge’s g) of 0.476, indicating a medium effect size favoring active stimulation over sham stimulation in the reduction of craving. No significant differences were found between the two brain stimulation techniques, even though their mechanisms of action are somewhat different [11, 19]. Regarding rTMS more specifically, a more recent systematic review, including 26 articles and 748 patients, showed significant reductions in cravings and substance use [20].

Even though these results are encouraging, the vast majority of these studies had small sample sizes and only short-term follow-up. In addition, there was considerable heterogeneity in terms of sample population, study design, and outcome measurements. A majority of these studies focused on clinical symptoms such as cravings instead of assessing more global and pertinent therapeutic effects such as reduced consumption or ability to maintain abstinence, which are the ultimate therapeutic goals for individuals with substance-related and addictive disorders.

We therefore propose to evaluate, for the first time and using a randomized controlled trial (RCT) involving a large sample, the clinical benefits of rTMS in patients with AUD who wish to reduce their alcohol consumption.

## Aims

Our hypothesis is that rTMS, which induces changes in the neuronal activity of the DLFPC that decrease cravings, can lead to a decrease in alcohol consumption in patients suffering from AUD.

Thus, the principal objective of this study is to evaluate, in non-abstinent patients with AUD, the efficacy of 1 week of rTMS (5 consecutive twice-daily sessions) versus placebo in reducing alcohol consumption within the 4 weeks following the treatment. In addition, we will repeat the alcohol consumption measurements periodically during 24 weeks of follow-up.

Moreover, we will assess the effects of rTMS on mood, cognitive behavior, and quality of life. In participants who have AUD combined with tobacco use disorder (TUD), the effect of rTMS on tobacco craving and consumption will be explored.

## Methods/design

### Overview

**Fig. 1:**
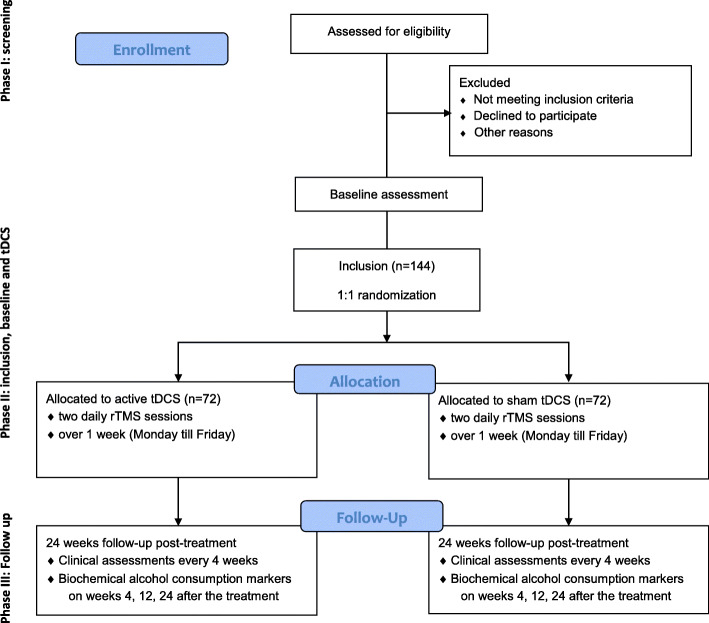
Study flow diagram

This is a multicenter, randomized, placebo-controlled, double-blind, parallel-group superiority study comparing 5 consecutive twice-daily sessions of active rTMS versus sham rTMS , with an allocation ratio in the trial of 1:1 (Fig. 1). The study is to be carried out in two French addictology departments (Dijon and Strasbourg), where patients with AUD are treated, and aims to recruit 144 patients with AUD over the course of 2 years. These departments provide consultation to a large number of patients, particularly for alcohol use disorder, among whom the study participants will be recruited. In addition, a communication via the social networks of the main center and through the local press is planned.

The study protocol was approved by an independent ethics committee (Committee for the Protection of Persons, West V) on 28 June 2020 under the number 2019-A03047-50 and by the French national agency for the safety of medical products and devices (*Agence National de Sécurité des Médicaments et des Produits de Santé*). After providing participants with a complete description of the study, written informed consent will be obtained from each participant.

Each revision will again be submitted to the ANSM and the ethics committee for approval. It will then be the subject of a complete re-editing of the protocol.

**Fig. 2:**
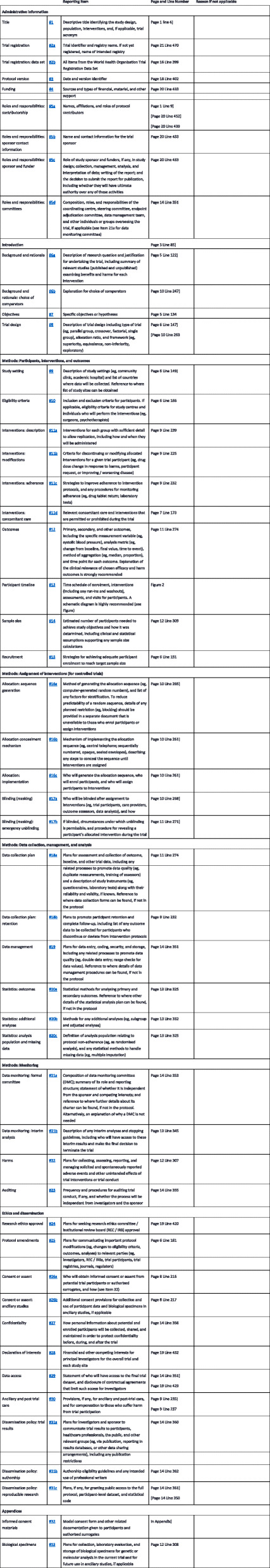
SPIRIT figure

The SPIRIT reporting guidelines were applied before submitting this manuscript [21]. The SPIRIT checklist is reported in Fig. 2.

### Inclusion criteria

Patients eligible to be enrolled in this trial: (1) males and females over 18 years of age, (2) meeting the criteria for mild to severe AUD as defined in the Diagnostic and Statistical Manual of Mental Disorders-5th edition (DSM-5) [12], (3) wishing to reduce their alcohol consumption, (4) and having experienced at least one prior attempt to achieve abstinence (unsuccessful or relapse).

### Exclusion criteria

Patients will be excluded if they have, at the inclusion visit, any of the following: (1) breath-alcohol concentration (BAC) > 0 mg per liter of exhaled air; (2) less than 6 heavy drinking days (HDD) in the previous 4 weeks (defined as more than 60 g of pure alcohol in men and 40 g in women consumed in 1 day) [1]; (3) average alcohol consumption below the medium risk level according to World health Organization (WHO) in the previous 4 weeks (≤ 40 g/day for men; ≤ 20 g/day for women) [22]; (4) more than 3 days of abstinence prior to inclusion; (5) a revised Clinical Institute Withdrawal Assessment (CIWA) for Alcohol score ≥ 10 (indicating the need for medication-supported detoxification); (6) concomitant treatment with disulfiram, acamprosate, topiramate, baclofene, naltrexone, or nalmefene; (7) a history of pre-delirium tremens and delirium tremens; (8) DSM-5 substance use disorder other than alcohol or tobacco use disorder; (9) acute psychiatric disorders that have required hospitalization and/or immediate adjustment of psychotropic medications; (10) severe major depression, as defined by 17-item Hamilton Depression scale (HAM-D) ≥ 24 [23]; (11) recent change in psychotropic medication (< 1 month); (12) severe chronic psychiatric disorders including schizophrenia, paranoia, and bipolar disorder types I and II; (13) advanced liver, kidney, cardiac, or pulmonary disease or other acute serious or unstable medical conditions that would compromise a patient’s participation in the study according to the physician’s judgment; (14) contra-indications to rTMS: personal history of convulsive seizures, cerebral vascular accident, pacemaker, neurosurgical clips, carotid or aortic clips, heart valves, hearing aid, ventricular bypass valve, sutures with wires or staples, foreign objects in the eye, shrapnel, other prosthesis, or intracranial ferromagnetic material; (15) women who are pregnant or lactating; (16) women of childbearing potential with a positive urine β–human chorionic gonadotrophin pregnancy test at inclusion; (17) concurrent participation in another trial, employees of the investigator or trial site, and patients protected by law; (18) persons who are not covered by national health insurance; (19) patients, in the opinion of the investigation, not able to complete the TimeLine Follow-Back (TLFB) and to record their daily alcohol consumption in a diary (derived from the TLFB) during the 3 months of the study; and (20) patients who refused to sign the consent form and “safety agreement”.

The “safety agreement” is a paper contract which expressly mentions that if a participant comes to the hospital for a visit or a rTMS session using his/her own car and has a BAC > 0.25 mg/l of exhaled air (which prohibits a person from driving a car in France), he/she will accept to give his/her car keys to the medical staff and authorize the staff to call a member of his/her family or a friend to take the patient home if he/she is unable to use public transport.

### Study process

The study will have three phases (Fig. 1):

1) During the first phase, subjects will be screened using the inclusion and exclusion criteria. Each subject will be given information regarding the implementation of the study and the objectives of the research.

2) The second phase will correspond to both the inclusion of participants and the period of rTMS sessions. This phase will always begin on Monday with 5 steps: (1) participants will be evaluated for study eligibility based on the inclusion and exclusion criteria; (2) after receiving the study information (including information concerning biological samples collected for biomarker assessment) again, they will have to sign the informed consent form (including the completion of the biological samples) and the “safety agreement” after ascertaining that participants have a zero alcohol blood level using breath alcohol concentration, collected by the investigators; (3) a clinical (including TLFB) and biological baseline assessment will be performed (visit 1); (4) included participants will be randomized to active or placebo rTMS; and (5) the first rTMS session will be delivered.

Then, daily sessions will be performed during the following days up to Friday, delivered by a trained operator. Since the treatment lasts only 5 consecutive days, no criteria were defined for discontinuing or modifying allocated intervention. In consideration of these elements, no other specific strategy to improve adherence to the protocol or its monitoring has been planned. The second phase will end by a clinical assessment (visit 2) once the last rTMS session has been delivered.

3) The third phase will be a 6-month follow-up phase without treatment. A clinical assessment will be performed every 4 weeks, and biochemical markers of alcohol consumption will be measured at 1, 3, and 6 months after the end of the stimulation. To promote participant retention and complete follow-up, we will use phone call to reschedule visits if they have been missed, or by default made them by phone.

The management of the patients at the end of the study will depend on the therapeutic results: it will be proposed to follow the reduction strategy in cases of efficacy, or to change the therapeutic goal through abstinence if alcohol consumption has worsened. In addition, no compensation is planned for to those who suffer harm from trial participation.

### Interventions

Patients will receive two consecutive 10 Hz rTMS sessions per day over the right DLPFC on 5 consecutive days. One rTMS session will consist of twenty trains of 50 pulses with an intensity of 110% of the resting motor threshold, separated by 19 intertrains of 30 s (1000 pulses per session). The two daily rTMS sessions will be separated by an interval of 15 min. This scheme of 2 sessions per day with a free interval of 15 min was successfully tested in a study on the treatment of depression [24]. In sum, each patient will receive 2000 pulses per day, and so 10,000 pulses at the end of the week of treatment.

The comparator will be sham rTMS. A placebo-controlled design was chosen as it is easy to apply with rTMS (placebo coil) and because placebo is a gold standard for randomized controlled trial. Moreover, the main aim of this study is not to compare rTMS to reference treatment, but to provide evidence of its efficacy.

We will use a Medtronic MagPro X100 Stimulator (MagVenture) with two types of coils: an MCF-B65 butterfly type coil with external liquid cooling unit (maximum magnetic field on the coil surface of 2.5 teslas) and a MCF-P-B65 sham coil with a magnetic field attenuation of more than 80%. After the randomization, following the arm to which the patient will be assigned (active stimulation or placebo), the operator will install the corresponding coil before the patient enters the room. During the stimulations, the investigators will not have access to stimulation room, only the operators will be able to attend the sessions, to preserve double-blinding.

### Randomization

Randomization will be performed the day of inclusion, online, using the secure CleanWeb^TM^ system by the investigator after identification though a personal password after a final check of the eligibility criteria (website https://chu-dijon.tentelemed.com). Patients will be randomly assigned to one of the two groups in a 1:1 ratio to one of two arms: rTMS active or rTMS placebo. The allocation algorithm, which relies on a minimization approach, is established by the study statistician (Unité de Soutien Méthodologique à la Recherche, USMR, Hospital University of Dijon) before the start of the trial. This allocation is stratified on center and sex. Investigators and patients will be blinded to the treatment assignment. A comprehensive document describing the randomization procedure will be kept in a confidential manner at the USMR.

Emergency unblinding will be performed in an emergency situation when the status of the brain stimulation (real or placebo) must be known to the investigator in order to provide appropriate medical treatment.

### Outcomes

In the “*Guideline on the development of medicinal products for the treatment of alcohol dependence*” for alcohol reduction strategies, the European Medicines Agency (EMA) recommend using a co-primary efficacy outcome, change from baseline in total alcohol consumption (TAC) per month, and reduction in number of HDD [1]. Thus, our principal effective criteria on alcohol reduction will be both the change in TAC from baseline to week 4, defined as mean daily alcohol consumption over 28 days (in g/day), and the number of HDD. Baseline will be defined as alcohol consumption during the 28 days before randomization using the *alcohol Timeline Followback* (TLFB) method, a validated method that retrospectively obtains estimates of daily drinking using a calendar [25, 26]. During follow-up, patients will be asked to report their alcohol consumption on a daily basis.

The secondary evaluation criteria will be the change from baseline to the end of the rTMS sessions, and then for each 4-week period after the treatment up to week 24 in:

- TAC (g/day) and number of HDD
- Proportion of subjects with a significant categorical shift in World Health Organization (WHO) risk levels of drinking: low risk (H≤40 g/d; F≤20 g/d), medium risk (H≤60 g/d; F≤40 g/d), high risk (H≤100 g/d; F≤60 g/d, and very high risk (H> 100 g/d; F> 60 g/d) [22]
- Proportion of subjects with a 50%, 70%, and 90% reduction in alcohol consumption as well as the proportion of patients who potentially achieve abstinence
- Level of alcohol dependence severity (alcohol dependence scale)
- Craving/urge to drink assessment (visual analogue scale, obsessive compulsive drinking scale)
- Clinical global impression-severity and improvement
- Scores for depression scales (HAM-D—17 items)
- Quality of Life (short form health survey—12 items)

Other secondary evaluation criteria will include the change from baseline at week 4, week 12, and week 24 after the treatment, in:

- Biochemical alcohol consumption markers (gamma glutamyl transferase, mean corpuscular volume, aspartate aminotransferase, alanine aminotransferase, and carbohydrate deficient transferrin)
- Cognitive assessment (Montreal cognitive assessment)
- Number of cigarettes smoked/day and craving for tobacco (visual analogue scale, tobacco craving questionnaire) for smokers.

Number of patients with adverse events will be determined at the time of each visit.

No genetic or molecular analysis is planned.

### Sample size calculation

The sample size was calculated using PASS software (version 11, Kayesville, UT, USA) [27]. As the primary efficacy outcome, the EMA recommend using both TAC and the number of HDD [1]. Besides, any reduction in total alcohol consumption of at least 10 g/day for patients with alcohol use disorders will reduce the annual and lifetime risk of alcohol-related death [28].

In this context, the sample size calculation based on an expected difference between the treatment groups of 15 g/day in total alcohol consumption or 3 days per month, with a standard deviation for the TAC of 30 g/day and with an autocorrelation of 0.3 to 0.7 between observations in the same subject, between 82 and 122 patients, would be required.

Considering a significance level of 1.25% (co-primary outcome) and a power of 80%, and with the hypothesis of a premature withdrawal or a non-initiation of treatment (acute repeated alcoholism) for 20% of individuals, 144 patients (72 per group) should be included to meet the objectives of the study.

### Statistical analysis

All randomized participants will be analyzed regardless of the treatment allocated (intention to treat analyses (ITT)) and after excluding patients with deviation from the protocol (per-protocol analyses (PP)).

The main analysis will be carried out with the intention to treat population. The co-primary outcome of change from baseline in TAC and reduction in the number of HDD at 4 weeks after treatment and its association with rTMS will be analyzed under the intention-to-treat principle using a mixed model for repeated measures.

The baseline and demographic characteristics of the two groups (active rTMS vs sham rTMS) will be described in terms of numbers and percentages and quantitative variables in means and standard deviations, or medians and interquartile intervals. The comparability of the two groups at baseline will be evaluated using the chi-squared test or Fisher’s test for qualitative variables, and Student’s T test or the Wilcoxon-Mann-Whitney test for continuous variables. Then, a mixed model repeated measures will be conducted to estimate the effect of treatment on TAC first, and HDD second. Observed cases will be considered random effects, and site, sex, time, and treatment as fixed effects. We will use multiple imputation techniques to compensate for potential bias introduced by missing endpoint data [29].

For the primary outcome, per-protocol analysis will also be conducted among participants who have completed baseline and endpoint assessments. The secondary outcome measurements will be analyzed with similar models to those used for the co-primary, continuous variables and logistic regression for dichotomized outcomes.

No interim analysis is planned.

All tests will be one-sided. The primary results will be examined at a significance level of 0.0125 (co-primary outcome). For secondary outcomes, the threshold for significance will be fixed at 0.025. All analyses will be performed using SAS version 9.4 (SAS Institute Inc) by the team of statisticians of the Unité de Soutien Méthodologique, Direction of Clinical Research, University Hospital of Dijon, France. Statistical code will not be made public.

Data management (data entry, coding, security and storage) and statistical evaluation will be carried out by the Clinical Research Department of the Dijon University Hospital, which is a service which is independent of the care units where the patients will be treated. As this is a very low risk trial for patients, French legislation does not require the creation and monitoring of a data monitoring committee. In consequence, no auditing of trial conduct was planned.

Only the patient’s anonymous code is reported in the case report form (CRF). This is comprised of the patient’s initials (first letter of the family name and the first name), the number of the center, and the number corresponding to the position in the list of inclusions.

## Results

The results will first be posted on the registration page of clinicaltrials.gov.

Then, we will communicate the results through oral communications, posters, and scientific publications. No publication restrictions are planned. To be considered an author, a person must (Vancouver rules):

- Make substantial contributions to the conception or design of the work; or the acquisition, analysis, or interpretation of data for the work;

- and to the drafting the work or revising it critically for important intellectual content;

- and to the final approval of the version to be published;

- and agree to be accountable for all aspects of the work in ensuring that questions related to the accuracy or integrity of any part of the work are appropriately investigated and resolved.

## Discussion

In recent years, rTMS was transformed from an emerging experimentation to a useful modern tool for the treatment of various neurologic and psychiatric disorders [30]. Some studies report beneficial effects for treating patients with substance-related and addictive disorders because it has beneficial effects on craving reduction and other cognitive dysfunctions that may underlie addictive disorders, with rTMS targeting the DLPFC [13, 16, 31–36]. Among the existing studies, only three have focused on patients treated for AUD, and these studies used an abstinence-based strategy. They found interesting results in terms of craving reduction, modulation of decision-making processes, and improvements in quality of life, and one of them observed a reduction in alcohol relapses [13, 34, 35]. However, none considered an approach based on reducing alcohol consumption even though a number of benefits can be expected, such as a reduction in alcohol-related damage and better acceptability of the therapeutic goal [9]. This strategy could be even more attractive given that treatment time is short (i.e., a few days of brain stimulation) and rTMS is known to be safe [37–39].

Although the current literature status seems to indicate rTMS may be effective in AUD and other substance use disorders, these conclusions needed to be further explored, mainly because these studies are limited by their small sample sizes, which has resulted in great heterogeneity, even in a well-conducted meta-analysis. Our aim is thus to conduct an RCT with a large sample size in order to investigate whether a treatment strategy using rTMS has the potential to become a promising treatment in AUD. In addition, unlike previous studies, our RCT will provide insight into whether rTMS has long-lasting effects in patients with AUD. We hope that our study will remedy these shortcomings and provide a high level of evidence for the short- and long-term efficacy of modulating DLPFC excitability via rTMS to treat AUD. This trial, which is to date one of the largest RCTs to assess the efficacy of rTMS in substance-related and addictive disorders, may also contribute to enhancing the efficacy of non-invasive brain stimulation techniques in the comprehensive treatment of addiction.

## Trial registration data

**Table:** 

Primary registry and trial identifying number	Clinicaltrials.gov NCT04773691
Date of registration in primary registry	February 26^th^, 2021
Source(s) of monetary or material support	National Hospital Research Program 2014 of the French Ministry of Health
Primary sponsor	National Hospital Research Program 2014 of the French Ministry of Health
Secondary sponsor	N/A
Contact for public queries	Dr Benjamin Petit, M.D., benjamin.petit@chu-dijon.fr
Contact for scientific queries	Dr Benjamin Petit, M.D., benjamin.petit@chu-dijon.fr
Public title	Efficacy of repetitive transcranial magnetic stimulation (rTMS) for reducing consumption in patients with alcohol use disorders (ALCOSTIM): study protocol for a randomized controlled trial
Scientific title	Efficacy of repetitive transcranial magnetic stimulation (rTMS) for reducing consumption in patients with alcohol use disorders (ALCOSTIM): study protocol for a randomized controlled trial
Countries of recruitment	France
Health condition(s) or problem(s) studied	Alcohol use disorder
Intervention(s)	Active rTMS (1,000 pulses per session, 2 sessions per day, 5 days)
	Sham rTMS
Key inclusion and exclusion criteria	Ages eligible for study: ≥ 18 years Genders eligible for study: both Accepts healthy volunteers: no
	Inclusion criteria: meeting the criteria for mild to severe AUD as defined in the Diagnostic and Statistical Manual of Mental Disorders-5th edition (DSM-5); wishing to reduce their alcohol consumption; and having experienced at least one prior attempt to achieve abstinence (unsuccessful or relapse).
	Exclusion criteria: - person who is not affiliated to or not a beneficiary of national health insurance - person subject to a legal protection measure (curatorship, guardianship) - person subject to a legal safeguard measure - pregnant, parturient or breastfeeding women - adult unable to express consent - patient of childbearing age with a positive pregnancy test at inclusion - patient with an exhaled alcohol level > 0 mg/l inclusive - patient with heavy alcohol consumption < 6 days in the 4 weeks prior to inclusion (European Medicine Agency, 2010; one day with alcohol consumption of 60 g or more for men and 40 g for women) - patient with an average alcohol consumption below the WHO average risk level in the 4 weeks prior to inclusion (WHO, 2000, less than or equal to 40 g/day for men and 40 g for women) - patient being abstinent more than 5 days before inclusion - patient with a CIWA (Clinical Institute Withdrawal Evaluation: assessment of the severity of alcohol withdrawal) score greater than or equal to 10 at inclusion - Patient with concomitant treatment with disulfiram, acamprosate, topiramate, baclofen, naltrexone, and nalmefen (< 1 month) - Patient with a history or presence of pre-delirium tremens or delirium tremens - Patient with a substance use disorder (DSM-5 criteria) with psychoactive substances other than tobacco and alcohol. - Patient with acute psychiatric disorders requiring hospitalization and/or immediate adjustment of psychotropic medication - Patient with severe depression, defined by a score of 24 or more on the Hamilton Depression Scale (HAM-D). - Patient who has had a recent change (< 1 month) in the prescription of psychotropic treatment - Patient with severe and/or chronic psychiatric disorders, including schizophrenia, paranoia and bipolar disorders type I and II - Patient with severe heart, kidney, liver or lung failure or other condition that the doctor believes could compromise the patient's participation in the study. - Patient with a contraindication to the practice of rTMS; personal history of seizure, pacemaker, neurosurgical clips, carotid or aortic clips, heart valves, hearing aid, ventricular bypass valve, sutures with wires or staples, foreign bodies in the eye, shrapnel, other prosthesis or cephalic ferromagnetic material. - Patient simultaneously participating in another therapeutic trial - Patient employed by the investigator or trial site - Patient who, according to the investigator, is unable to complete a consumption diary and follow up visits for 6 months - Patient refusing to sign the "safety contract "* specific to the study
Study type	Interventional
	Allocation: randomized intervention model. Parallel assignment. Masking: double blind (patient, investigator)
	Primary purpose: treatment
Date of first enrolment	March 2021
Target sample size	144
Recruitment status	Recruiting
Primary outcome(s)	change in TAC and number of HDD from baseline to week 4
Key secondary outcomes	change from baseline to the end of the rTMS sessions, and then for multiple interim endpoints after the treatment up to week 24: - TAC (g/day) and number of HDD - Proportion of subjects with a significant categorical shift in World Health Organization (WHO) risk levels of drinking: low risk (H≤40 g/d; F≤20 g/d), medium risk (H≤60 g/d; F≤40 g/d), high risk (H≤100 g/d; F≤60 g/d, and very high risk (H> 100 g/d; F> 60 g/d) [22] - Proportion of subjects with a 50%, 70% and 90% reduction in alcohol consumption as well as the proportion of patients who potentially achieve abstinence - Level of alcohol dependence severity (alcohol dependence scale) - Craving/urge to drink assessment (visual analogue scale, obsessive compulsive drinking scale) - Clinical global impression-severity and improvement - Scores for depression scales (HAM-D – 17 items) - Quality of Life (short form health survey – 12 items) - Biochemical alcohol consumption markers (gamma glutamyl transferase, mean corpuscular volume, aspartate aminotransferase, alanine aminotransferase and carbohydrate deficient transferrin) - Cognitive assessment (Montreal cognitive assessment) - Number of cigarettes smoked/day and craving for tobacco (visual analogue scale, tobacco craving questionnaire) for smokers.

## Trial status

Version 1.1; last edited 22/06/2020

Enrolment for this study will begin on March 1, 2021. At the time of submission, we have enrolled the first participant.

Study duration as predicted: 30 months and 1 week

## Data Availability

The datasets analyzed during the current study are available from the corresponding author on reasonable request.

## Abbreviations

AUD: Alcohol use disorders
CIWA: Clinical institute withdrawal assessment for alcohol
CRF: Case report form
DALYs: Disability-adjusted life years
DLPFC: Dorsolateral prefrontal cortex
DSM-5: Diagnostic and statistical manual of mental disorders. 5th ed
EMA: European medicines agency
HAM-D: Hamilton depression rating scale
HDD: Heavy drinking days
rTMS: Repetitive transcranial magnetic stimulation
RCT: Randomized controlled trial
TAC: Total alcohol consumption
tDCS: Transcranial direct current stimulation
TLFB: Alcohol timeline followback
TUD: Tobacco use disorder
WHO: World Health Organization
